# Preventing HIV infection in pregnancy: a comprehensive ANC-based intervention in Western Uganda

**DOI:** 10.1093/eurpub/ckac129.391

**Published:** 2022-10-25

**Authors:** S Theuring, LS Jahn, A Kengonzi, SN Kabwama, J Rubaihayo

**Affiliations:** 1 Institute of Tropical Medicine and International Health, Charité- Universitätsmedizin, Berlin, Germany; 2 School of Health Sciences, Mountains of the Moon University, Fort Portal, Uganda; 3 School of Public Health, Makerere University, Kampala, Uganda

## Abstract

**Introduction:**

Pregnant women in sub-Saharan Africa represent a high-risk group for HIV infection, but most endemic countries including Uganda do not engage specific HIV prevention measures in pregnancy. This longitudinal study aimed to assess outcomes of a comprehensive, ANC-embedded strategy to prevent seroconversions during pregnancy in Western Uganda.

**Methods:**

HIV-negative ANC clients were administered an HIV risk assessment tool, followed by individual risk counselling. They received a fixed appointment for repeat HIV testing after three months. Those attending ANC without partners obtained formal partner invitation letters. At follow-up after three months, women not attending repeat testing were reminded via text message. Post-intervention risk behavior engagement was captured. We analyzed uptake of the intervention, HIV incidence rate, and associations with risk behavior.

**Results:**

Of 1081 participants, 116 (10.7%) reported risk behavior engagement at first ANC visit, 148/1081 (13.7%) were accompanied by partners. The repeat test visit was attended by 848/1081 (78.5%) women, 42 (5.0%, p < 0.001) reported post-intervention risk behavior engagement, and 248 (29.4%, p < 0.001) were accompanied by partners. Seroconversion occurred in two women. In multivariable logistic regression, rural facility clients compared to urban ones (aOR 3.96; 95%CI 1.53-10.26), and women with positive or unknown partner HIV-status (aOR 2.86; 1.18-6.91) and partner alcohol abuse (aOR 2.68; 1.15-6.26) had increased odds for engagement in risk behavior despite the intervention.

**Conclusions:**

After our intervention, risk behavior in pregnancy was reduced by half, and partner attendance had doubled compared to baseline. Our cohort showed a 0.76/100 women-years HIV incidence rate compared to 2.85 in pre-intervention data from the same setting. Clients from rural settings and women experiencing precarious partner situations require special attention to reduce risk behavior engagement during pregnancy.

**Key messages:**

• HIV incidence in pregnancy in Western Uganda can be significantly reduced through a comprehensive, ANC-based counselling intervention.

• Pregnant women from rural settings and those experiencing precarious partner situations require special attention regarding sexual risk behavior.

